# Editorial: Innate Immune Cell Determinants of T Cell Immunity: From Basic Mechanisms to Clinical Implications

**DOI:** 10.3389/fimmu.2015.00664

**Published:** 2016-01-14

**Authors:** Elisabetta Padovan, Stefan F. Martin

**Affiliations:** ^1^Department of Biomedicine, Institute of Surgical Research, Basel University Hospital, University of Basel, Basel, Switzerland; ^2^Allergy Research Group, Department of Dermatology, Medical Center, University of Freiburg, Freiburg, Germany

**Keywords:** innate immunity, T cell memory, antigen presentation, vaccines, human diseases

**The Editorial on the Research Topic**

**Innate Immune Cell Determinants of T Cell Immunity: From Basic Mechanisms to Clinical Implications**

Long-lasting T cell immunity is delivered by individual T lymphocytes expressing clonally distributed antigen-specific receptors. Following priming in lymph nodes, naïve CD4^+^ and CD8^+^ T lymphocytes proliferate and generate clones of effector cells that deliver unique effector functions in peripheral tissues. Moreover, long-lasting memory T cells generated during priming are rapidly engaged upon reexposure to the same antigen, even years after their primary induction. Notably, this very efficient protection system cannot unfold without accessory cells. Our frontiers research topic features different innate immune cell subsets and the crucial roles they play in the initiation and maintenance of T cell immunity. By comprehensively describing positive and negative outcomes of these events, the contributions provide a meaningful link between basic findings and clinical applications.

## T Cell Physiology Directed by Innate Immune Cells

Following the seminal discovery of Steinman and Cohn in 1973 (1, 2) describing a rare cell type initiating antigen-specific responses, dendritic cells (DC) have taken up the stage for several decades as professional antigen-presenting cells (APC). In their reviews, Geginat et al. and Clausen and Stoitzner dissect the instrumental role played by specialized DC subsets in instructing protective T cell immunity, emphasizing how this specialization, conserved in mice and humans, suits at best the need of dedicated and qualitative different “classes” of T cells for immune homeostasis, defense against pathogens, and responses to vaccines and allergens.

Dendritic cells, however, do not stand alone in this process. Indeed, although DC activated through pattern-recognition receptors (PRR) are competent for CD4^+^ T cell priming, they require feedback from other T cell subsets, including iNKT, γδT, and CD4^+^ T helper (Th) cells, for the generation of antigen-specific CD8^+^ T cell immunity. iNKT cells and γδT cells are innate-like T cell subsets that recognize lipid and metabolites in a non-MHC-restricted fashion. The contribution of Salio and Cerundolo highlights the specific characteristics of these cell types and their modality of activation by different tissue-resident APC, focusing on the intracellular pathways that regulate lipid and metabolite Ag presentation at steady state and upon infection. The role of these cells in “licensing” DC for CD8^+^ T cell priming is illustrated by Gottschalk et al., presenting a comparative functional analysis of DC licensed by iNKT and Th cells.

Immune responses to infections and other assaults are initiated in the target tissues. These do not only harbor DC but also other immune cell subtypes that are either tissue resident or become recruited. Activation of innate immune cells, such as mast cells (MC) and neutrophils, will most likely influence the activation and polarization of DC, for example, the pattern of cytokines expressed by the DC. Thereby, these cells may indirectly influence the polarization of naïve T cells by DC in the lymph node. In addition, neutrophils have been shown to migrate to lymph nodes, where they may directly contribute to T cell priming.

Secondary activation is also influenced by innate immune cell subsets. For instance, the early phase of infection is characterized by a rapid recruitment of neutrophils and monocytes into the inflamed tissue, where these phagocytes colocalize with tissue-resident memory T cells. In the most recent years, consistent evidences have accumulated in support of the capacity of these accessory cells to influence T cell immunity *in vivo*. The contributions of Leliefeld et al. and Roberts et al. address the role of, respectively, neutrophils and monocytes as “bystander activators” that favor survival and activation of T cells, independently of TCR antigen specificity. Notably, both cell types can also act as APC delivering Ag-specific and costimulatory signals to T cells, and their collaborative endeavors were found to positively and negatively modulate the activity of different effector T cell subsets, including conventional and innate-like T cells. Moreover, neutrophils and monocytes may differentiate and acquire different functional programs in response to signals provided by activated T cells and influence the quality of T cell responses even at later stages of infections and malignant transformation.

At barrier sites T cell responses become modulated also by the activity of tissue-resident MC, basophils, and innate lymphoid cells (ILC) through their bidirectional interaction with T cells.

Basophils and MC, originally regarded as “degranulating” inflammatory cells rapidly responding to the triggering of PRR, are now recognized to participate in the regulation of T cell immunity. The contributions of Sarfati et al. and Bulfone-Paus and Bahri feature the capacity of these two cell subsets to skew naïve T cell priming and modulate effector T cell responses by acting as cytokine-secreting bystanders in coordination with DC, as well as by physically interacting with T lymphocytes. The availability of suitable animal models, described by Otsuka and Kabashima, has increased our understanding of basophils as putative specialized APC for low-molecular-weight compounds, as compared to MC, although these findings await confirmation for human basophils.

Intriguingly, the effect of basophils and MC on the tissue microenvironment affects also the survival and activity of ILC. This is a heterogeneous family of rare immune cells, comprehensively described in the review of Von Burg et al., influencing T cell responses through Ag-independent modulation of T cell survival and proliferation, as well as MHC-restricted Ag presentation. Interestingly, these functions, which are required for the maintenance of immune homeostasis, are segregated among different ILC types and compartmentalized in different anatomical locations. Notably, the interaction of ILC with T cells is also bidirectional, since ILC survival and proliferation requires cytokines released by activated T cells.

## Natural Immune Deficiencies Deranging Human T Cell Immunity

The unfolding of Ag-specific T cell immunity and their regulation critically depends on the formation of highly organized intercellular junctions between APC and T cells, referred to as immune synapses (IS). The contribution of Kallikourdis et al. features the functions of these intercellular structures by illustrating the consequences ensuing from inborn errors of their structural components. Notably, IS instability caused by missing or dysfunctional components affects trafficking and functions of both APC and T cells and associate to autoimmunity and recurrent infections.

## Detrimental Consequences of Deranged T Cell Immunity at Different Body Sites and Immune Intervention

Although the actions of innate immune cells fulfills the need of initiating and maintaining protective T cell responses, the excessive presence or activity of individual determinants may be detrimental to the host, because it could promote tissue destruction as in autoimmunity and allergy, or conversely, prevent the induction of protective immune responses against malignant tissues. The capacity of neutrophils, monocytes, and basophils to acquire different functional profiles in response to environmental triggers and also their role as “bystander activators,” can indeed become detrimental, as underscored by the role of basophils and MC in allergies and chronic inflammation (Sarfati et al.; Bulfone-Paus and Bahri), of monocyte/macrophages in rheumatoid arthritis (Roberts et al.) and of neutrophils and DC in cancer (Clausen and Stoitzner; Leliefeld et al.). Based on this knowledge, it is possible to consider the possibility of exploiting different types of DC licensing for inhibiting autoimmunity or enhancing protective immunity against pathogens and tumors (Salio and Cerundolo; Gottschalk et al.). In line with this, Martin et al. expand on the promising finding that anticancer drugs act as APC modulators in antitumor immunity.

In a totally new vision, Biedermann et al. comprehensively describe the causal link between skin commensals and atopic dermatitis, highlighting how activation of skin-resident DC and myeloid-derived suppressor cells by T cell-derived cytokines and microbial components exacerbates atopic dermatitis and the clinical benefits induced by therapeutic alteration of the skin microbiome.

## Author Contributions

EP and SM edited and reviewed this topic.

## Conflict of Interest Statement

The authors declare that the work was conducted in the absence of any commercial or financial relationships that could be construed as a potential conflict of interest.
